# Trajectories of vital signs in patients with COVID-19

**DOI:** 10.1016/j.resuscitation.2020.09.002

**Published:** 2020-11

**Authors:** Marco A.F. Pimentel, Oliver C. Redfern, Robert Hatch, J. Duncan Young, Lionel Tarassenko, Peter J. Watkinson

**Affiliations:** a Institute of Biomedical Engineering, Department of Engineering Science, University of Oxford, Oxford, UK; b Nuffield Department of Clinical Neurosciences, University of Oxford, Oxford, UK

## Abstract

**Background:**

The global pandemic of coronavirus disease 2019 (COVID-19) has placed a huge strain on UK hospitals. Early studies suggest that patients can deteriorate quickly after admission to hospital. The aim of this study was to model changes in vital signs for patients hospitalised with COVID-19.

**Methods:**

This was a retrospective observational study of adult patients with COVID-19 admitted to one acute hospital trust in the UK (CV) and a cohort of patients admitted to the same hospital between 2013-2017 with viral pneumonia (VI). The primary outcome was the start of continuous positive airway pressure/non-invasive positive pressure ventilation, ICU admission or death in hospital. We used non-linear mixed-effects models to compare changes in vital sign observations prior to the primary outcome. Using observations and FiO_2_ measured at discharge in the VI cohort as the model of normality, we also combined individual vital signs into a single novelty score.

**Results:**

There were 497 cases of COVID-19, of whom 373 had been discharged from hospital. 135 (36.2%) of patients experienced the primary outcome, of whom 99 died in hospital. In-hospital mortality was over 4-times higher in the CV than the VI cohort (26.5% vs 6%). For those patients who experienced the primary outcome, CV patients became increasingly hypoxaemic, with a median estimated FiO_2_ (0.75) higher than that of the VI cohort (estimated FiO_2_ of 0.35). Prior to the primary outcome, blood pressure remained within normal range, and there was only a small rise in heart rate. The novelty score showed that patients with COVID-19 deteriorated more rapidly that patients with viral pneumonia.

**Conclusions:**

Patients with COVID-19 who deteriorate in hospital experience rapidly-worsening respiratory failure, with low SpO_2_ and high FiO_2_, but only minor abnormalities in other vital signs. This has potential implications for the ability of early warning scores to identify deteriorating patients.

## Take-home message

This study demonstrates that patients with COVID-19 deteriorate more rapidly than seen in other viral pneumonias, with progressively lower oxygen saturations, greater oxygen requirements and only minor abnormalities in other vital signs. The national early warning score 2 (NEWS2) does not account for the degree of supplemental oxygen, suggesting that early warning systems could be enhanced by accounting for the degree of oxygen usage for patients with COVID-19.

## Background

The global pandemic of coronavirus disease 2019 (COVID-19) caused by severe acute respiratory syndrome coronavirus 2 (SARS-CoV-2) has placed a huge strain on UK hospitals.^1^ UK Government strategy recognises that intensive care unit (ICU) capacity is a limited, yet ICUs are a critical resource in the treatment of COVID-19.^2^ Current evidence suggests that almost all patients admitted to the ICU will require respiratory support, of whom 60-90% will receive mechanical ventilation.^3^, ^4^ Around one third of patients will also require advanced cardiovascular or renal support.^4^, ^5^ However, data from the Intensive Care National Audit and Research Centre (ICNARC) estimates that mortality in patients admitted to ICU could exceed 50%.^4^ Several observational studies have described factors that could affect prognosis,^3^, ^6^, ^7^, ^8^, ^9^, ^10^, ^11^ although most are either small (< 1000 cases) or limited to patients who were treated in the ICU.

It is likely that most patients with COVID-19 will not be admitted to ICU, although they remain at risk of deterioration after admission to hospital. The Royal College of Physicians have recommended the continued use of the National Early Warning Score (NEWS2) to identify deterioration from vital sign observations.^12^ However, they acknowledge that NEWS2 is limited by not accounting for the degree of oxygen supplementation. We are unaware of any studies that have examined changes in vital signs in patients with COVID-19 during their hospital stay.

In this study, we describe the trajectories of individual vital signs of the first 373 patients presenting with COVID-19 to a UK teaching hospital. We then combine individual vital signs into a single “novelty score”, to explore overall physiological derangement. We compare trajectories, using a cohort of patients admitted to the same hospital with viral pneumonia, prior to the emergence of the COVID-19 pandemic.

## Methods

Data were extracted from the Hospital Alerting Via Electronic Noticeboard (HAVEN) study. This is an ongoing project funded by the Wellcome Trust, the Department of Health and Oxford Biomedical Research Centre, which uses routinely collected data from the electronic health care records (EHR) to improve the detection of deteriorating patients. The study is reported in line with the TRIPOD statement.^13^

### Ethics

Health Research Authority approval was obtained for gathering the data used in this study from the South Central Oxford C Research Ethics Committee (16/SC/0264) and Confidentiality Advisory Group (16/CAG/0066).

### Data sources and linkage

The study database included: electronic recordings of vital signs (from the System for Electronic Notification and Documentation, SEND),^14^ laboratory test results, patient demographics and timing of in-hospital deaths and unanticipated ICU admissions. The data were purged of all direct identifiers and pseudonymised prior to analysis by the research team.

### Study design and setting

This was a retrospective cohort study conducted in the Oxford University NHS Hospitals Trust. The Trust comprises four hospitals: John Radcliffe Hospital (university hospital), Horton General Hospital (district general hospital), Churchill hospital (university cancer centre) and the Nuffield Orthopaedic Hospital (tertiary orthopaedic centre).

### Participants and sample size

This study uses data from two patient cohorts.

The “COVID-19” cohort (CV) included patients admitted to the study hospitals from 13^th^ March 2020 to 28^th^ April 2020 with a positive COVID-19 RT-PCR test. The “viral pneumonia” (VI) cohort included patients admitted with viral pneumonia to the study hospitals for the years 2013 to 2017. Admissions for viral pneumonia were identified using ICD-10 coding, where the primary diagnosis was either Influenza due to identified zoonotic or pandemic influenza virus (J09); Influenza due to identified seasonal influenza virus (J10); or viral pneumonia, not elsewhere classified (J12).

We excluded admissions in both cohorts where: the patient was < 16 years of age or where no vital signs were recorded outside of the ICU.

The sample size was determined by the number of admissions available to the research team at the time of analysis.

### Outcomes

The primary outcome was a composite of: the start of continuous positive airway pressure (CPAP) or non-invasive positive pressure ventilation (NIPPV/NIV), admission to ICU or death in hospital. The secondary outcome was a composite of ICU admission or death in hospital.

### Variables

For each patient admission, we extracted the following vital signs: systolic and diastolic blood pressure (BP), temperature, peripheral oxygen saturations (SpO_2_), heart rate and respiratory rate. We also estimated the fraction of inspired oxygen (eFiO_2_) from the recorded oxygen mask type and flow rate using a previously described method.^15^, ^16^ From this, we calculated the ratio of SpO_2_ to eFiO_2_.

### Statistical analysis

Patient demographics and other clinical information were summarised as follows: for continuous variables we used the median/interquartile range, and for binary or categorical variables we used the proportion. Confidence intervals for proportions were calculated using the Pearson-Klopper method.

#### Trajectory of individual vital signs

We modelled changes in individual vital signs (which we refer to as trajectories) over the course of hospital admission by modelling changes in the population. We fitted separate models for each physiological variable (systolic and diastolic blood pressure, heart rate, SpO_2_, temperature, respiratory rate, FiO_2_ and SpO_2_-to-eFiO_2_ ratio) to estimate the distribution (median and interquartile range, or the 25^th^, 50^th^, and 75^th^ centiles) dependent on the time to the primary outcome or discharge from hospital. For the event group, which included patients who experienced the primary outcome, we used the time to the event as the covariate in our models; where multiple events occurred for a given patient, we took the first. For the non-event group (i.e., patients who did not experience the primary outcome), we used the time to discharge from hospital. We presented centiles for the seven days that preceded the event/discharge for each vital sign graphically.

All time-dependent distributions were modelled with the gamlss package^17^ using methods described previously.^18^ We chose the optimal model by comparing the predicted with the empirical centiles for each vital sign (see Supplementary Material for more details).

We repeated the analysis described above for the secondary outcome.

#### Novelty score combining individual vital signs

In order to summarise the cumulative physiological abnormalities, we combined individual vital signs into a single novelty score. The development of the novelty score is described in detail in the Supplementary Material. Briefly, we used a previously published approach^19^, ^20^ to construct a multi-dimensional distribution of “normal” vital sign measurements, extracted from observations taken at hospital discharge in the VI cohort. The following variables were included in the novelty score: heart rate, respiratory rate, SpO_2_, temperature, systolic and diastolic blood pressure and eFiO_2_. After imputing missing data (see below), we compared each set of vital sign observations to the multi-dimensional distribution by calculating the negative log-likelihood.

For comparison, we also calculated the aggregated NEWS. We determined the NEWS for each set of vital signs, which included heart, rate, SpO_2_, temperature, systolic blood pressure, an indicator of whether the patient was given supplemental oxygen at the time of each measurement, and level of consciousness, typically measured using the Alert-Voice-Pain-Unresponsive (AVPU) scale. Where the patient’s conscious level had been assessed only using the Glasgow Coma Scale (GCS), we converted GCS to an AVPU equivalent.^15^

We calculated trajectories of both NEWS and the novelty score using the same method as for individual vital signs.

#### Missing data

For the analysis of individual vital signs, we made no attempt to impute missing data. Prior to calculating the novelty score for a set of vital sign observations, we imputed individual vital signs by carrying forward the previous measurement. Where no previous measurement was available, we used the mean value calculated from discharge observations in the VI cohort (see above).

## Results

### COVID-19 (CV) and Viral pneumonia (VI) cohorts

There were 497 cases in of COVID-19, of which 381 had been admitted and subsequently discharged from hospital. Of those, eight patients with no recorded vital signs were excluded, leaving 373 completed admissions in the CV cohort. After exclusion criteria were applied, there were 485 patients in the viral pneumonia cohort (VI). Table 1 describes both cohorts. In-hospital mortality was over 4 times higher in the CV than the VI cohort (26.5% 95% CI [22.1-31.3] vs 6% [4.0-8.5]) with 65.7% of deaths occurring in male patients (see Table S1, in Supplementary Material). The percentage of patients experiencing the primary composite outcome in the CV cohort was nearly three-fold higher (36.2% [31.3-41.3] vs 12.8% [9.9-16.1]). In the CV cohort, 53% died without transfer to the ICU, or receiving CPAP or NIPPV/NIV. In both cohorts, patients who died were older than survivors (CV median age 82 vs 65; VI 86 vs 72).

**Table 1:** Demographics of COVID-19 (CV) and Viral Infections cohorts. IQR: interquartile range; CPAP: continuous positive airway pressure; NIPPV/NIV: non-invasive positive pressure ventilation. ICU: intensive care unit.

	CV cohort	VI cohort
Admissions included in the study, N	373	485
Age at admission, median [IQR]	72 [57–82]	73 [57–84]
Gender (male), N (%)	209 (56.0)	224 (46.2)
Emergency admissions, N (%)	369 (98.9)	483 (99.6)
In-hospital deaths, N (%)	99 (26.5)	29 (6.0)
ICU admissions, N (%)	36 (9.7)	25 (5.2)
CPAP or NIPPV/NIV, N (%)	29 (7.8)	13 (2.7)
Primary composite outcome, N (%)	135 (36.2)	62 (12.8)
Time to start of CPAP or NIPPV/NIV in days, median [IQR]	2.2 [1.2–3.4]	2.4 [1.5–3.4]
Time to admission to ICU in days, median [IQR]	1.1 [0.6–2.1]	0.9 [0.2–1.9]
Time to in-hospital death in days, median [IQR]	5.5 [3.2–8.5]	11.3 [7.4–15.9]

### Vital sign trajectories

**Fig. 1:**
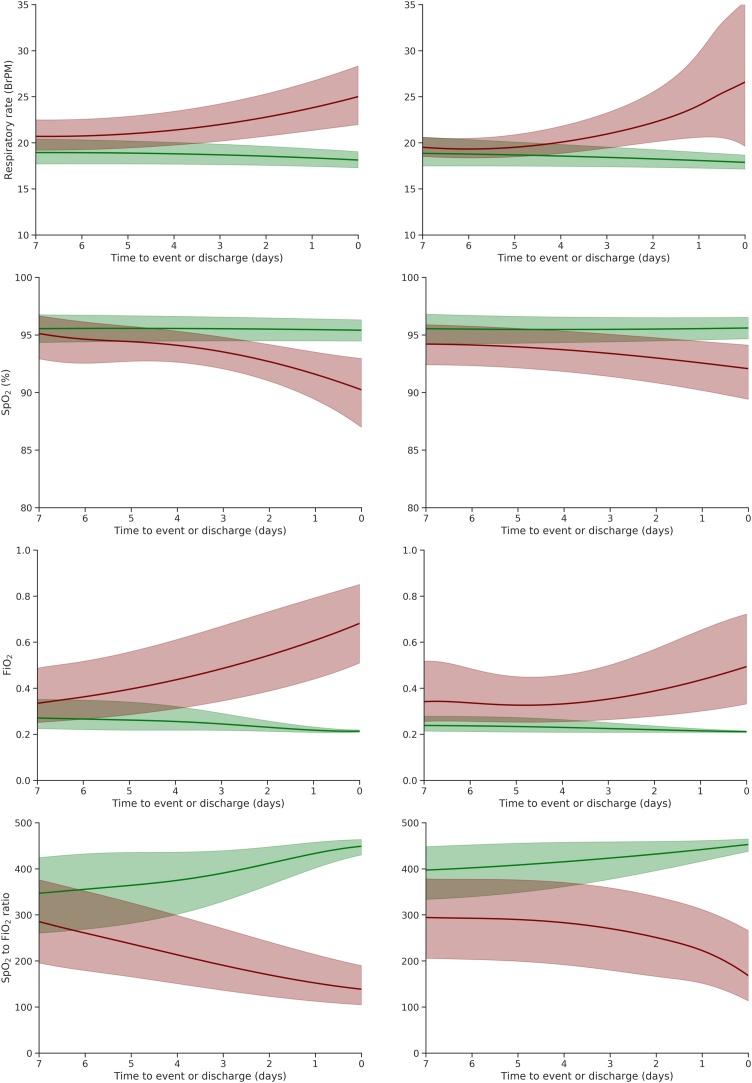
Trajectories of respiratory vital signs for the CV (left) and VI (right) cohorts in the 7 days prior to the primary outcome (red) and prior to discharge from hospital alive (green). Lines correspond to the median trajectories and shaded areas correspond to the interquartile range.

Fig. 1 shows the trajectories of respiratory vital signs for patients in the CV and VI cohorts prior to either the primary outcome (event group) or discharge from hospital. In both cohorts, there was a substantial increase in respiratory rate and supplemental oxygen (estimated FiO_2_) in the five days prior to the primary outcome, with a concomitant decrease in SpO_2_ and the SpO_2_/FiO_2_ ratio. In the CV cohort, the median eFiO_2_ rose from 0.35 to 0.75 prior to event, with a much smaller increase seen in the VI cohort (0.35 to 0.50). Median SpO_2_ in the VI cohort remained broadly within normal range (>93%) up to the time of the event. In contrast, median SpO_2_ in the CV cohort dropped below 93% one day prior to the event, despite an accompanying increase in eFiO_2_.

**Fig. 2:**
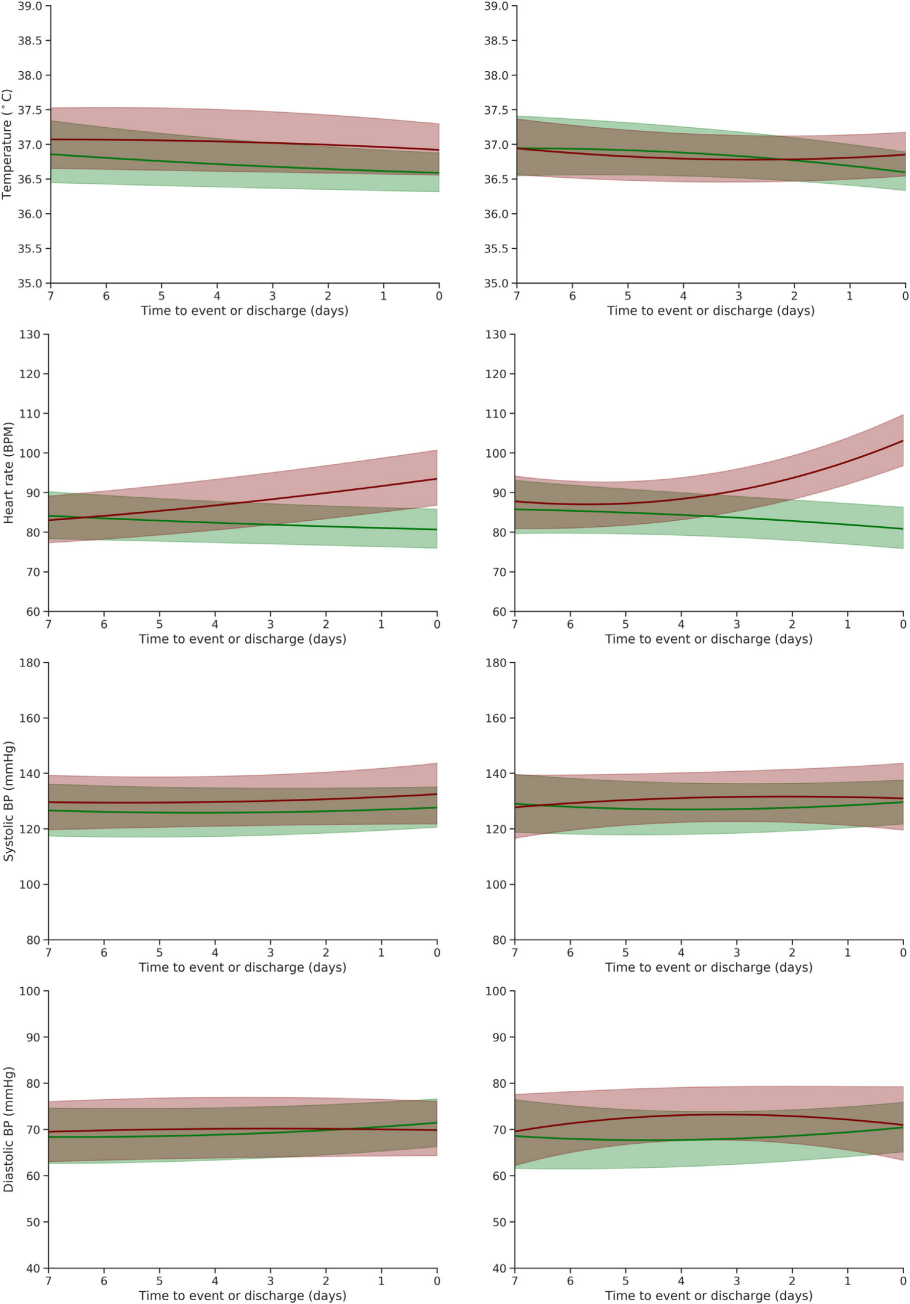
Trajectories of other vital signs for the CV (left) and VI (right) cohorts in the 7 days prior to the primary outcome (red) and prior to discharge from hospital alive (green). Lines correspond to the median trajectories and shaded areas correspond to the interquartile range.

For patients who experienced the primary outcome, median temperature (Fig. 2) was similar in both cohorts (showing little variation from around 37.0 °C). There was an increase in heart rate in the three days prior to event, although this rise was steeper in the VI cohort. Blood pressure measurements (systolic and diastolic) remained within the normal range.

Trajectories for the secondary outcome were similar (Figures S1 & S2).

### Novelty score trajectories

**Fig. 3:**
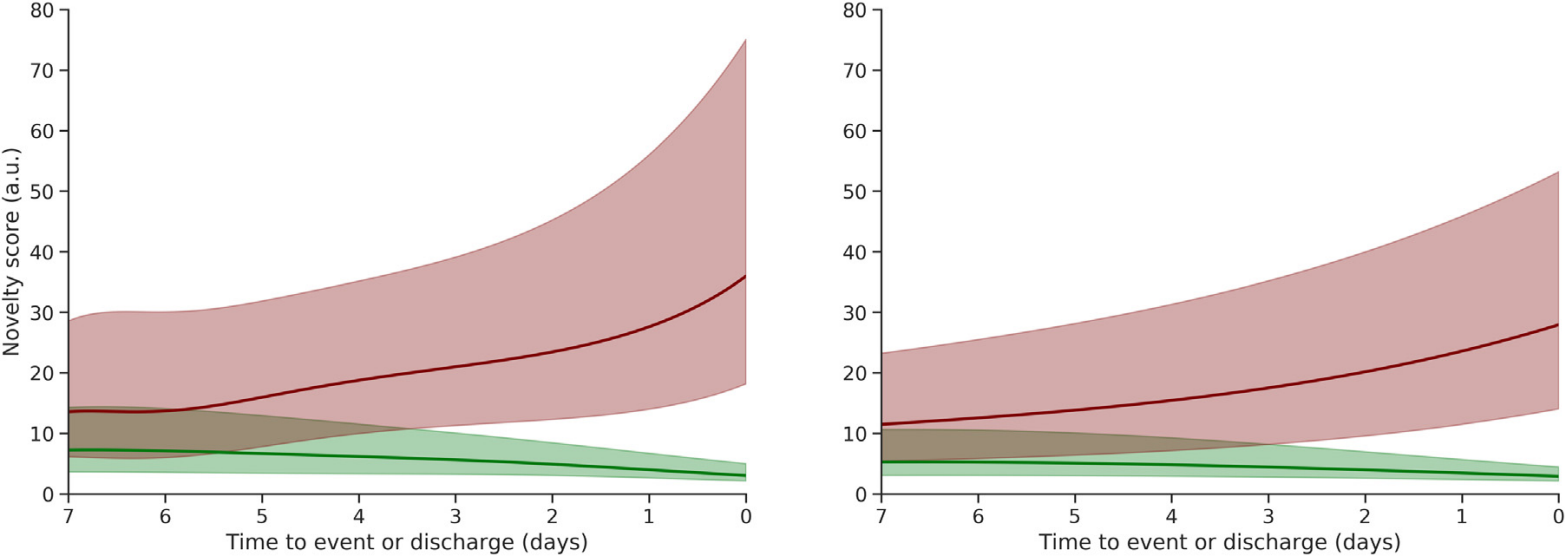
Trajectories of both NEWS (a) and the novelty score (b) for the CV (left) and VI (right) cohorts in the 7 days prior to the primary outcome (red) and prior to discharge from hospital alive (green). Lines correspond to the median trajectories and shaded areas correspond to the interquartile range.

Fig. 3b shows the trajectory of the novelty score, which combines individual vital sign abnormalities and eFiO_2_ into a single value. The median novelty score begins to increase prior to the event in both cohorts, but both the rate and magnitude of the rise are greater in the CV cohort (as also shown in Table S3). A further comparison of the trajectory of the novelty score and NEWS is provided is Figure S4.

## Discussion

### Main findings

In this retrospective study of 373 COVID-19 patients admitted to an acute hospital in the UK, nearly 36% required advanced respiratory support or died. Patients who died were older and more likely to be male. Mortality was four times higher than for patients admitted with viral pneumonia prior to the COVID-19 pandemic.

Patients in the COVID-19 (CV) cohort who experienced the primary outcome were increasingly tachypnoeic and required increasing amounts of supplemental oxygen. On average, these patients had a small increase in heart rate, but no marked change in blood pressure or temperature. These results are consistent with a previous study from China where tachycardia and hypotension were uncommon features,^9^ Changes in vital signs were broadly similar to those observed in the VI cohort. However, on average, patients with COVID-19 received double the levels of supplemental oxygen. Despite more intensive oxygen therapy, their recorded oxygen saturations became lower as they deteriorated. This could be because clinicians tolerated lower oxygen saturations in COVID-19 patients or it may be a limitation of the use of a composite outcome. 53% of patients in the CV cohort died without CPAP, NIPPV/NIV or admission to ICU, compared to 44% of VI patients. Consequently, the decrease in oxygen saturations might be due to the trajectory being more heavily weighted by data from patients approaching the end of life.

Trajectories of both NEWS and the novelty score showed that patients with COVID-19 exhibited greater physiological derangement as they deteriorated than those in the VI cohort. It is worth noting that our novelty score was developed as a purely summary metric, with as few prior assumptions as possible. The score reflects the deviation of each vital sign from their values in a “normal” dataset (see Supplementary Material). Our analyses did not include an assessment of the novelty score’s ability to identify deteriorating patients. Nevertheless, it does address a recognised limitation of NEWS2^12^, ^21^ by accounting for the degree of oxygen supplementation. Excluding estimated FiO_2_ (see Supplementary Material - Figure S3) from the novelty score showed a reduced difference in the rate of deterioration between the CV and VI cohorts.

Comparing the performance of the novelty score to NEWS was outside of the scope of this study, however the trajectories of the novelty score in the COVID cohort demonstrate a more rapid change in the last 24 hours before the primary outcome and a greater dynamic range during this period. In contrast, NEWS has a much slower rate of rise towards its alerting threshold throughout the admission, which could make detection of deterioration more challenging (Figure S4). This suggests that adapting early warning scores, such as NEWS2, to account for the amount of supplemental oxygen could improve their performance in patients with COVID-19.

Early warning scores were developed to help clinicians identify patients at risk of deteriorating, to encourage timely escalation and remedial treatment. Unlike bacterial pneumonia (for example), there are currently no effective treatments for COVID-19 except supportive care. For this reason, identifying deteriorating patients earlier might be less likely to improve their outcome. However, both frontline clinicians^22^ and professional bodies^23^ have recognised that, due to the speed at which COVID-19 can progress (as shown in our results), earlier identification of patients with limited prognosis could allow more time to discuss the risks/benefits of advanced respiratory support. As the volume of data available on patients with COVID-19 remains limited at present, we would echo the note of caution sounded by Wynants et al in their review of new proposed prognostic scores.^24^

In addition, we note that risk scoring systems such as NEWS were developed to support decision-making around escalation of care, and provide a means of communicating clinical acuity between clinical staff and across different healthcare organisations.^12^ Hence, they provide a tool that should be used as an aid to clinical assessment, but should never replace clinical concern (regardless of the score).

### Limitations

In this study, we investigated the trajectories of all main vital signs that are used as components of most early warning scores. Nevertheless, we did not include the trajectory of the patients’ level of consciousness in our analysis, which is one of the main components of NEWS. The level of consciousness, as given by the AVPU scale, is a categorical variable (rather than a numeric variable) with four levels, which makes it difficult to determine and visualise its trajectory. From Table S3 (see Supplemental Materials), we note, however, that the level of consciousness is unlikely to have a significant contribution to the score, as the proportion of patients not in an “alert state” does not increase throughout the days leading up to the event in either cohort.

Moreover, we note that this study was conducted in a single UK hospital Trust and was limited by the relatively small number of COVID-19 cases available at the time of analysis. We chose to exclude 116 patients who were still in hospital in order to reduce the bias in our analysis. Our conclusions must therefore be tempered by these limitations and should be externally validated in larger cohorts of patients hospitalised with COVID-19.

## Conclusions

This study shows that patients with COVID-19 who deteriorate in hospital experience rapidly-worsening respiratory failure, with low SpO_2_ and high eFiO_2_, but only minor abnormalities in other vital signs. This has potential implications for the ability of early warning scores to identify deteriorating patients.

## CRediT author statement

Marco Pimentel performed the analysis and co-authored the paper.

Oliver Redfern performed the analysis, assisted with data extraction and co-authored the paper.

Robert Hatch performed the data extraction, assisted in the analysis and co-authored the paper.

Duncan Young, Lionel Taraseenko and Peter Watkinson oversaw the research and revised the manuscript.

## Funding

This publication presents independent research commissioned by the Health Innovation Challenge Fund (HICF-R9-524; WT-103703/Z/ 14/Z), a funding partnership between the Department of Health & Social Care and Wellcome Trust. The views expressed in this publication are those of the authors and not necessarily those of the Department of Health or Wellcome Trust. PW and LT are also supported by the National Institute for Health Research, Biomedical Research Centre, Oxford. RH is supported by an NIHR Doctoral Research Fellowship.This research has been conducted using the Oxford University Hospitals NHS Foundation Trust Clinical Data Warehouse, which is supported by the NIHR Oxford Biomedical Research Centre and Oxford University Hospitals NHS Foundation Trust.

## Conflicts of interest

PW and LT report significant grants from the National Institute of Health Research (NIHR) and the NIHR Biomedical Research Centre, Oxford, during the conduct of the study. PW and LT report grants and personal fees from Sensyne Health, outside the submitted work. PW is a consultant to Sensyne Health and holds shares in the company. LT works part-time for Sensyne Health and holds share options in the company.
