# Supplementary material for: Trajectories of vital signs in patients with COVID-19
Source: Resuscitation. 2020 Nov;156:99–106. doi: 10.1016/j.resuscitation.2020.09.002 (PMC7481128; doi:10.1016/j.resuscitation.2020.09.002)
Supplement: Supplementary file 1 [file mmc1.docx]

# Trajectories of vital signs in patients with COVID-19

## Authors

Marco AF Pimentel^†^, Postdoctoral Researcher, Institute of Biomedical Engineering, Department of Engineering Science, University of Oxford, Oxford, UK

Oliver C Redfern^†^, Clinical Researcher, Nuffield Department of Clinical Neurosciences, University of Oxford, Oxford, UK

Robert Hatch, Clinical Research Fellow, Nuffield Department of Clinical Neurosciences, University of Oxford, Oxford, UK

J Duncan Young, Professor of Intensive Care Medicine, Nuffield Department of Clinical Neurosciences, University of Oxford, Oxford, UK

Lionel Tarassenko, Professor of Electrical Engineering, Institute of Biomedical Engineering, Department of Engineering Science, University of Oxford, Oxford UK

Peter J Watkinson, Associate Professor of Intensive Care Medicine, Nuffield Department of Clinical Neurosciences, University of Oxford, Oxford, UK

^†^ joint first authors

## Supplementary material

### Demographic details of cohorts

Tables S1 and S2 show futher demographic information for the CV and VI cohorts (N/A denotes “not applicable”).

Table S1. Demographics of COVID-19 (CV) cohort.

|  | All | Died | Survived |
| --- | --- | --- | --- |
| **Admissions, N** | 373 | 99 | 274 |
| **Age, median (IQR)** | 72 [57-82] | 82 [75-87] | 65 [52-78] |
| **Male, N (%)** | 209 (56.0) | 65 (65.7) | 144 (52.6) |
| **Emergency Admissions, N (%)** | 369 (98.9) | 99 (100.0) | 270 (98.5) |
| **Deaths, N (%)** | 99 (26.5) | 99 (100.0) | 0 (0.0) |
| **ICU admissions, N (%)** | 36 (9.7) | 8 (8.1) | 28 (10.2) |
| **CPAP or NIPPV/NIV, N (%)** | 29 (7.8) | 20 (20.2) | 9 (3.3) |
| **Primary composite outcome, N (%)** | 135 (36.2) | 99 (100.0) | 36 (13.1) |
| Time to start of CPAP or NIPPV/NIV in days, median [IQR] | 2.2 [1.2-3.4] | 2.1 [0.9-3.2] | 2.9 [1.5-3.9] |
| **Time to ICU admission in days, median [IQR]** | 1.1 [0.3-2.1] | 0.6 [0.3-1.7] | 1.1 [0.4-2.1] |
| **Time to in-hospital death in days, median [IQR]** | 5.5 [3.2-8.5] | 5.5 [3.2-8.5] | N/A |

Table S2. Demographics of viral pneumovia (VI) cohort.

|  | All | Died | Survived |
| --- | --- | --- | --- |
| **Admissions, N** | 485 | 29 | 456 |
| **Age, median (IQR)** | 73 [57-84] | 86 [81-89] | 72 [56-83] |
| **Male, N (%)** | 224 (46.2) | 15 (51.7) | 209 (45.8) |
| **Emergency Admissions** | 483 (99.6) | 29 (100.0) | 454 (99.6) |
| **Deaths, N (%)** | 29 (6.0) | 29 (100.0) | 0 (0.0) |
| **ICU admissions, N (%)** | 25 (5.2) | 1 (3.4) | 24 (5.3) |
| **CPAP or NIPPV/NIV, N (%)** | 13 (2.7) | 1 (3.4) | 12 (2.6) |
| **Composite primary outcome, N (%)** | 62 (12.8) | 29 (100.0) | 33 (7.2) |
| Time to start of CPAP or NIPPV/NIV in days, median (IQR) | 2.4 [1.5-3.4] | 2.2 [1.2-2.9] | 3.4 [3.2-4] |
| **Time to ICU admission in days, median (IQR)** | 0.9 [0.2-1.9] | 12.3 [12.3-12.3] | 0.8 [0.2-1.8] |
| **Time to in-hospital death in days, median (IQR)** | 11.3 [7.4-15.9] | 11.3 [7.4-15.9] | N/A |

### Statistical approach to confirming the best fit to the data

We used the lambda, mu, sigma and Box-Cox Cole and Green distribution method[1,2] to estimate smoothed centiles for each physiological variable as a function of the time to event of interest or discharge from hospital. Distribution parameters were modelled using second-order fractional polynomials (with the time to event as the indepedent covariate) and random effects terms (accounting for the correlation between repeated measurements on the same patient).

We assessed goodness of fit by visual inspection of the empirical centiles versus fitted centiles.

### Vital sign trajectories

Figures S1 and S2 show trajectories for individual vital signs for the secondary outcome (ICU admission or death).


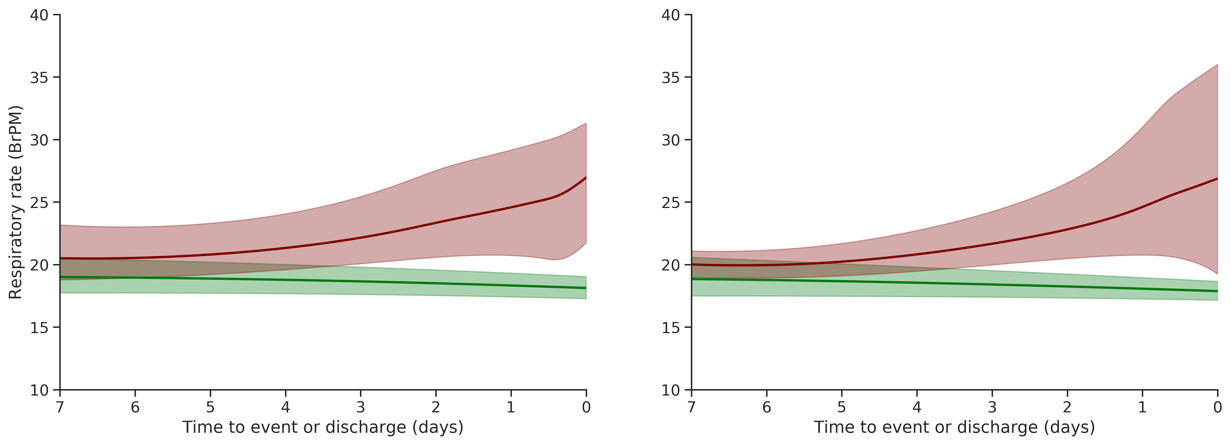

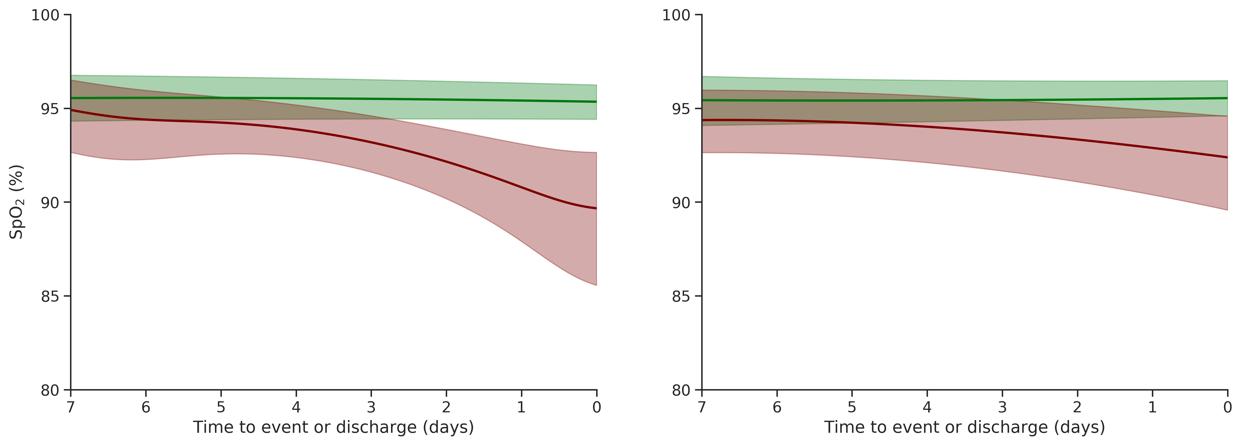


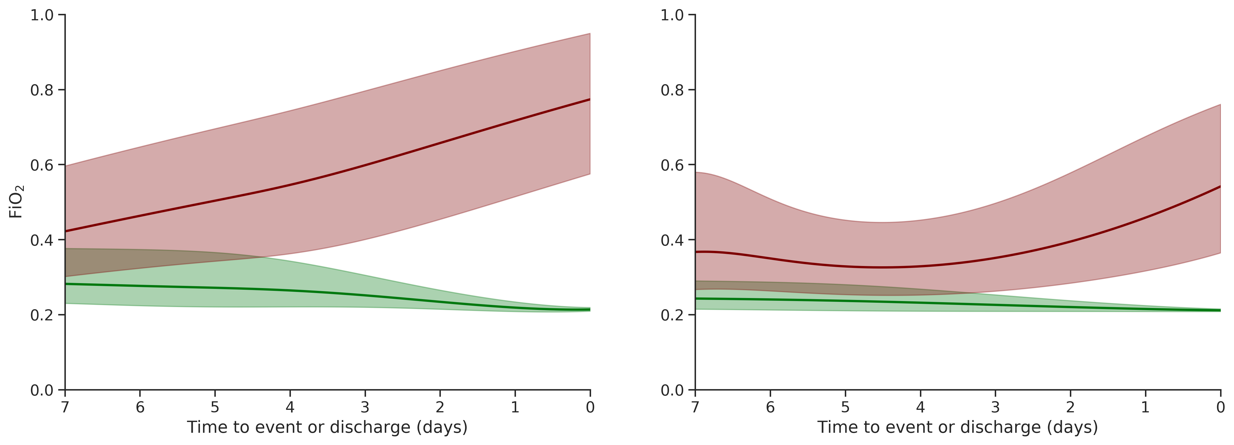

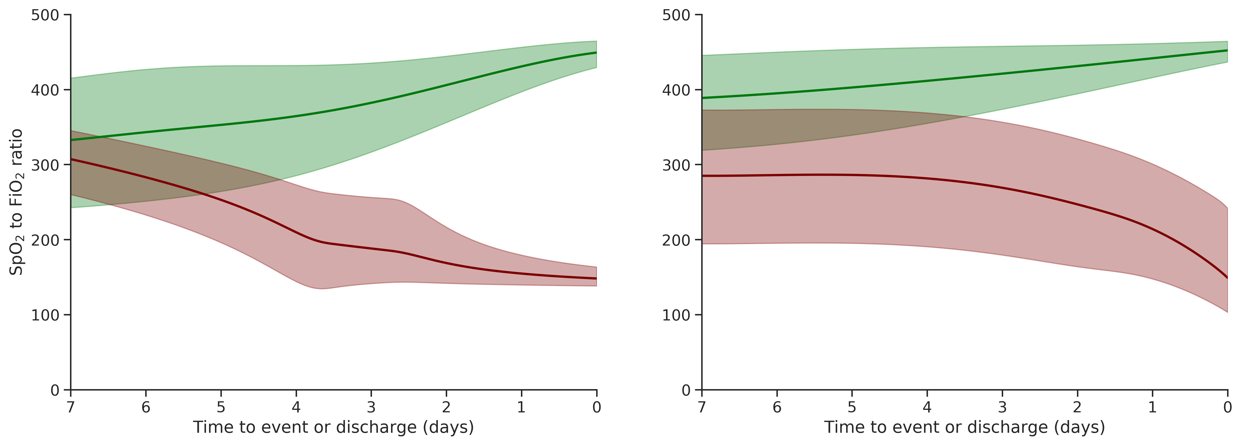


Figure S1. Trajectories of respiratory vital signs for the CV (left) and VI (right) cohorts in the 7 days prior to the primary outcome (red) and prior to discharge from hospital alive (green). Lines correspond to the median trajectories and shaded areas correspond to the interquartile range.


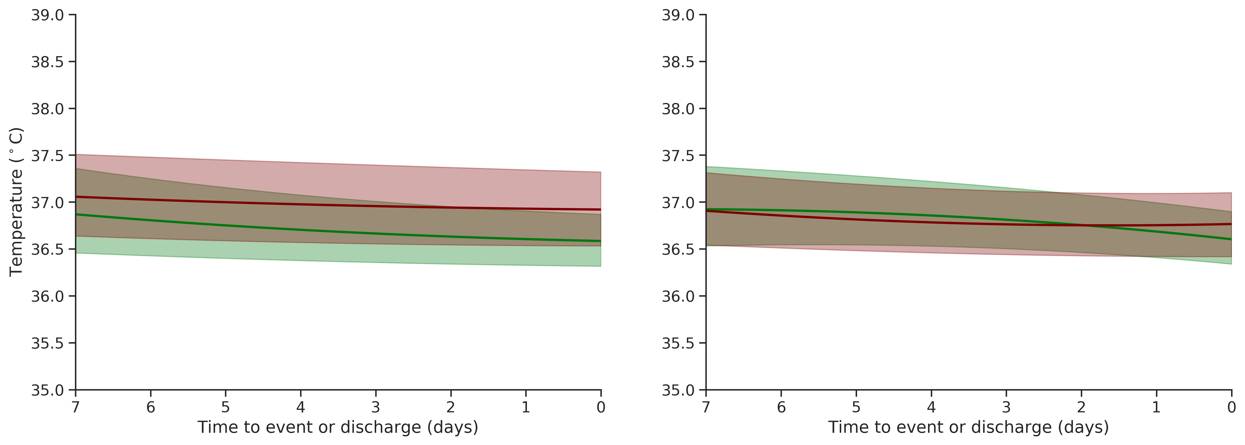

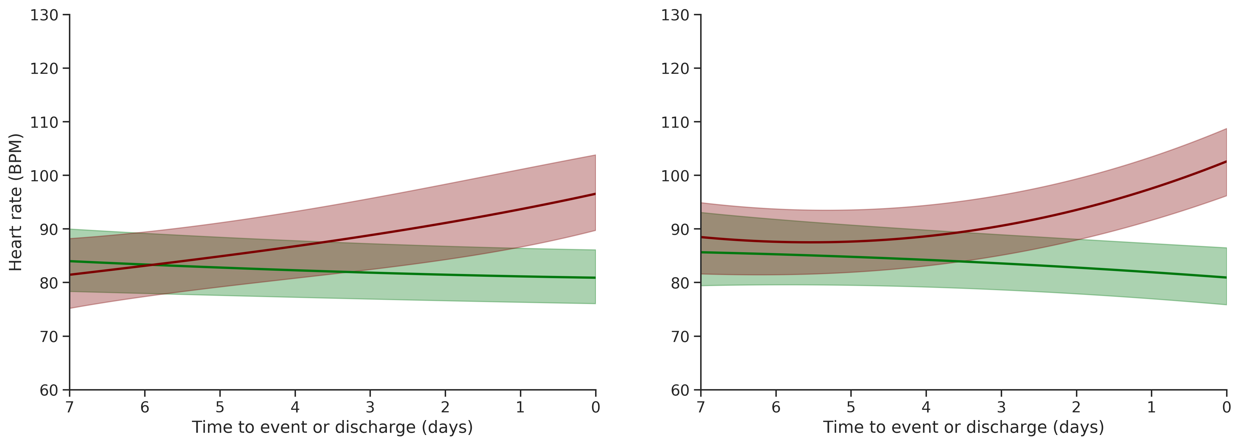

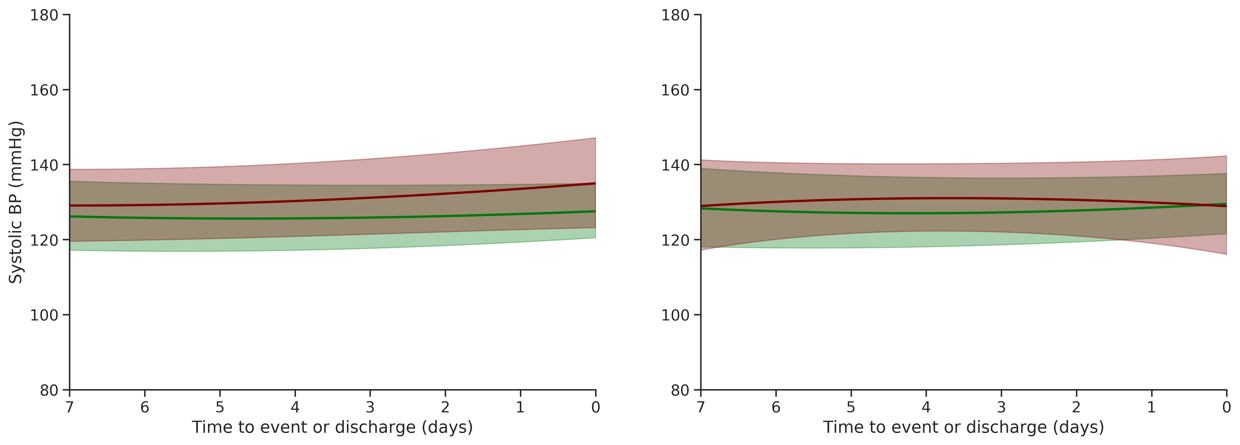

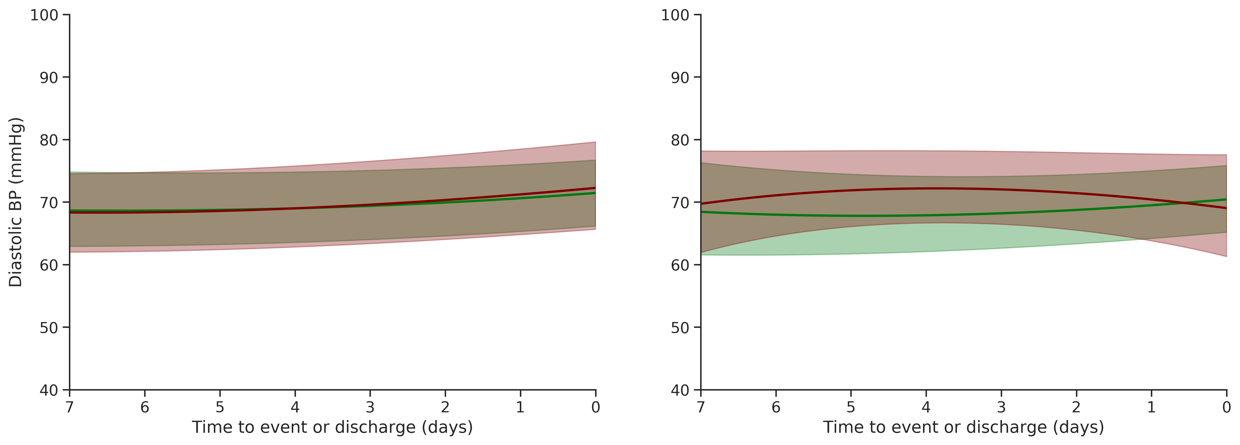


Figure S2. Trajectories of other vital signs for the CV (left) and VI (right) cohorts in the 7 days prior to the primary outcome (red) and prior to discharge from hospital alive (green). Lines correspond to the median trajectories and shaded areas correspond to the interquartile range.

### Novelty score and National Early Warning Score (NEWS) trajectories


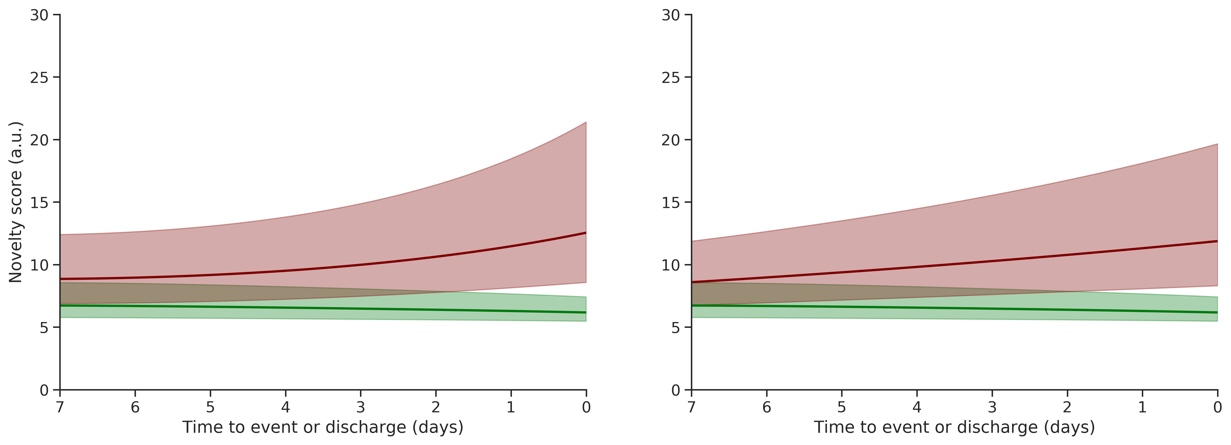


Figure S3. Trajectories of the novelty score (excluding FiO_2_) for the CV (left) and VI (right) cohorts in the 7 days prior to the primary outcome (red) and prior to discharge from hospital alive (green). Lines correspond to the median trajectories and shaded areas correspond to the interquartile range.


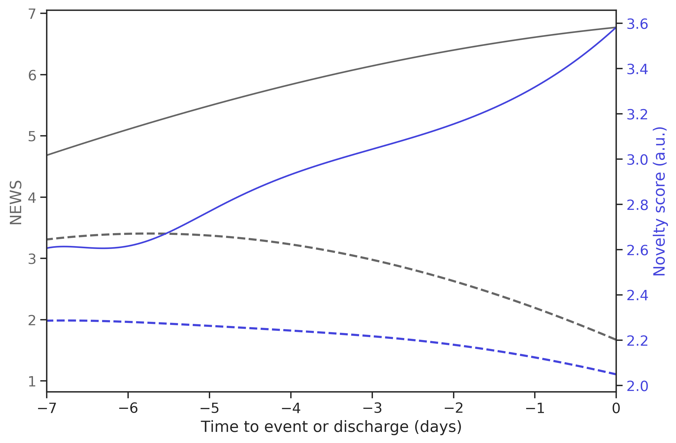

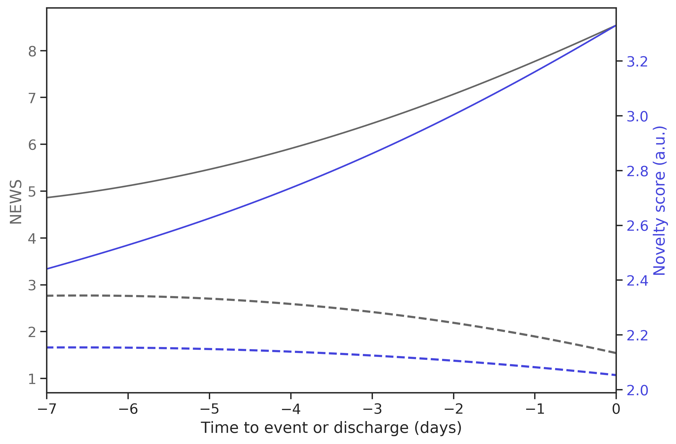


Figure S4. Trajectories of the novelty score (blue) vs NEWS (gray) for the CV (left) and VI (right) cohorts in the 7 days prior to the primary outcome (solid) and prior to discharge from hospital alive (broken line). Lines correspond to the median trajectories.

### Development of vital sign novelty score

#### Multidimensional model of “normal” vital signs

In order to aggregate and combine all predictor variables (or vital signs) into a single marker (or score), we considered the construction of a model of “normal” vital signs, as described in previous work[3,4]. For patients who were discharged alive from hospital in the VI cohort, we selected the last set of vital sign measurements taken in the 24 hours prior to discharge. The rationale for using these particular observations was to use the most physiologically stable data in the period of hospital admission, to which observations from other periods (and from other patients) may be compared. We calculated normalised histograms (unit area under the curve) for each of these vital signs: heart rate, respiratory rate, blood oxygen saturation (SpO_2_), (tympanic) temperature, and systolic and diastolic blood pressure (BP), and eFiO_2_.

We constructed a multidimensional model of “normality” using all discharge vital sign observations described above. To do this, we used a kernel density estimation (KDE) method to estimate a 7-dimensional probability density function (PDF) of the discharge vital signs. All variables were standardised using a zero-mean unit-variance transformation. We used a (isotropic) Gaussian kernel with 5-fold cross validation, to identify the bandwidth parameter that maximised the likelihood.

#### Novelty score and representing physiological trajectories

To represent the physiological trajectory for each cohort, we calculated the likelihood of each set of vital sign observations with respect to the model of normality. A high log-likelihood would indicate that a given set of vital sign observations was similar to the discharge vital signs used to generate the model of normality. Consequently, we used the negative log-likelihood as our “novelty score”, so that higher values indicate observations that are most different from those in the model of normality.

## References

[1] Cole TJ, Green PJ. Smoothing reference centile curves: The lms method and penalized likelihood. Stat Med 1992;11:1305–19. https://doi.org/10.1002/sim.4780111005.

[2] Rigby RA, Stasinopoulos DM. Using the Box-Cox t distribution in GAMLSS to model skewness and kurtosis. Stat Model An Int J 2006;6:209–29. https://doi.org/10.1191/1471082X06st122oa.

[3] Johnson AEW, Burgess J, Pimentel MAF, Clifton DA, Young JD, Watkinson PJ, et al. Physiological trajectory of patients pre and post ICU discharge^1^. 2014 36th Annu. Int. Conf. IEEE Eng. Med. Biol. Soc., vol. 2014, IEEE; 2014, p. 3160–3. https://doi.org/10.1109/EMBC.2014.6944293.

[4] Pimentel MAFF, Clifton DA, Clifton L, Watkinson PJ, Tarassenko L. Modelling physiological deterioration in post-operative patient vital-sign data. Med Biol Eng Comput 2013;51:869–77. https://doi.org/10.1007/s11517-013-1059-0.
